# Antimicrobial Activities of Three Medicinal Plants and Investigation of Flavonoids of *Tripleurospermum disciforme*

**Published:** 2015

**Authors:** Zahra Tofighi, Maryam Molazem, Behnaz Doostdar, Parisa Taban, Ahmad Reza Shahverdi, Nasrin Samadi, Narguess Yassa

**Affiliations:** a *Department of Pharmacognosy and Medicinal Plant Research Center,Faculty of Pharmacy, Tehran University of Medical Sciences, Tehran, Iran. *; b *Pharmaceutical Sciences Branch, Islamic Azad University, Tehran, Iran. *; c *Department of Pharmaceutical Biotechnology and Medical Nanotechnology Research Center,Faculty of Pharmacy, Tehran University of Medical Sciences, Tehran, Iran. *; d *Department of Drug and Food Control, Faculty of Pharmacy, Tehran University of Medical Sciences, Tehran, Iran.*

**Keywords:** *Securigera securidaca*, *Rosa damascena*, *Tripleurospermum disciforme*, antimicrobial activity, Flavonoids

## Abstract

*Rosa damascena*, *Tripleurospermum disciforme* and *Securigera securidaca* were used as disinfectant agents and for treatment of some disease in folk medicine of Iran. The antimicrobial effects of different fractions of seeds extract of *S. securidaca*, petals extract of *R. damascena *and aerial parts extract of *T. disciforme* were examined against some gram positive, gram negative and fungi by cup plate diffusion method. The petroleum ether and chloroform fractions of *S. securidaca* showed antibacterial activities against *Staphylococcus aureus *and *Pseudomonas aeruginosa*, while its methanol fraction had no antibacterial effects. *R. damascena *petals extract demonstrated antibacterial activities against* Bacillus cereus*,* Staphylococcus epidermidis, S. aureus *and *Pseudomonas aeruginosa*. *T. disciforme *aerial parts extract exhibited antimicrobial effects only against *S. aureus* and *S. epidermidis*. None of the fractions had any antifungal activities. Therefore, present study confirmed utility of these plants as disinfectant agents. Six flavonoids were isolated from *T. disciforme*: Luteolin, Quercetin-7-O-glucoside, Kaempferol, Kaempferol-7-O-glucoside, Apigenin and Apigenin-7-O-glucoside. The flavonoids and the antimicrobial activity of *T. disciforme* are reported for the first time.

## Introduction

There are growing interests in use of plants as natural antimicrobial agents because they do not induce antibiotic resistance which is common in the synthetic antibiotics*. *Securigera securidaca (L.) Deg. & Dorf. (Fabaceae) is one of three species of this genus which grows in Iran (1). *Rosa damascena* Mill. (Rosaceae) is a small plant with aromatic flower which appears in spring (2). Nowadays, *R. damascena* is the principle species cultivated for Rose water and attar in central part of Iran (Kashan), India and Bulgaria (3). *Tripleurospermum disciforme* (C.A. Mey) Schultz Bip., a genus of Asteraceae, is one of the native plants of Europe and western Asia (4). It was grown in many parts of Iran. These three plants had many traditional and folk uses in Iran but there were a few reports about antimicrobial effects of them. 

The people in the south of Iran used oral administration of the seeds of *S. securidaca* for hypoglycemic effects. *S. securidaca *extract significantly reduced glucose level in diabetic animals by a mechanism different from sulfonylurea agents (5, 6). 

*R. damascena *has some benefits such as cooling, soothing, astringent and anti inflammatory effects (7). Its extract and essential oil showed antioxidant and antibacterial properties (8-10). Rose water is a natural healer for various skin problems and a skin care in folk medicine of Iran. It is an important ingredient in many body creams and cosmetics in the world due to its pleasant fragrance and useful properties. 

*T. disciforme* was used as anti inflammatory, anti spasmodic, anti septic, carminative and as a hair color (11, 12). 

The objective of present research is to evaluate antimicrobial effects of *S. securidaca*, *R. damascena* and *T. disciforme* extracts and isolation and identification of compounds of *T. disciforme*.

## Experimental

*Plant material*

The seeds of *S. securidaca*, petals of *R. damascena *and top flowered of *T. disciforme* were collected in September, May and July 2011 around the Fars, Gilan and Tehran Provinces of Iran, respectively. The plants were dried in shade and powdered. A voucher specimen of each plant is deposited at Herbarium of Faculty of Pharmacy, Tehran University of Medical Sciences. 

*Preparation of extracts*

The powder of dried seeds of *S. securidaca***, **petals of *R. damascena* and top flowered of *T. disciforme *(400 g of each sample) were macerated separately with 80% methanol at room temperature in a percolator, then solvents concentrated in vacuum to give gummy residue (crude extract). The crude extract of *S. securidaca* was re-extracted with petroleum ether, chloroform and methanol to achieve different fractions. The concentrated extracts and fractions were kept at 4 ºC prior to antimicrobial tests. 

*Microorganisms and media*

The various organisms were used as standard strains in this study, include *Staphylococcus aureus* ATCC6538, *Staphylococcus epidermidis* ATCC12229, *Bacillus subtilis *ATCC6633 and *Bacillus cereus *ATCC1274 as Gram positive bacteria; *Pseudomonas aeruginosa* ATCC9027, *Escherichia coli* ATCC8739 and *Klebsiella pneumoniae* ATCC1003 as Gram negative bacteria; *Candida albicans* ATCC1023 and *Aspergillus niger* ATCC16404 as fungi, which were obtained from Department of Drug and Food Control, Faculty of Pharmacy, Tehran University of Medical Sciences. Soybean Casein Digest Agar (Merck, Germany) and Saburouad Dextrose Agar (Merck, Germany) were used as medium for the growth of bacterial and fungal strains, respectively.

*Antimicrobial assay*

The antibacterial and antifungal activity of the different extracts and fractions of the plants were studied by cup plate diffusion method as described by Warnock DW (13). Each organism was separately suspended in normal saline solution which was equal to 10^8^ CFU/mL. For preparing base plates, 25 mL of cooled media was poured in to the sterile Petri dishes and inoculated with one of the microorganisms by spreading microbial suspension over the plate with a sterile cotton swab. Then in each plate, holes of 7 mm in diameter were made at equal distances using sterile cork borer. Different concentrations of fractions (100, 50, 25, 12.5, 6.25, 3.125 and 1.562 mg/mL) were prepared and DMSO (dimethyl sulfoxide) with 1% w/v concentration was used as a solvent. 100 µL of each extracts and fractions were added to each hole on the medium. The plates containing bacteria and fungi were incubated at 35 ºC for 24 h and 25 ºC for 48 h, respectively. The diameter of zone of inhibition was measured in millimeters after incubation as an indication of activity and compared with the solvent as negative control. Gentamycin and Nystatin were used as positive control. All the tests were repeated in triplicate. 

*Elucidation of compounds of T. disciforme*

Since there was few reports about phytochemical investigation of *T. disciforme *extract, it was selected for isolation and purification of compounds. Crude extract (313.61 g) from 1.5 Kg of *T. disciforme* was fractionated with petrol ether (PE) and chloroform (CH) yield 50.11 and 13.5 g respectively. Remaining gummy residue which was soluble in methanol called methanol fraction (ME; 250 g).

ME fraction (5 g) was applied to reverse phase silicagel column chromatography (2.5×13.5 cm) and eluted with gradient mobile phase H_2_O-MeOH (80:20 → 0: 100, V/V) to afford 5 subfractions. M_3_ subfraction (564 mg) was selected for size exclusion chromatography (SEC) on Sephadex LH-20 column (2.1×67 cm) eluted with MeOH. Compounds 1 (5.5 mg), 2 (4.3 mg) and 3 (12 mg) were isolated and purified. M_4 _subfraction (435 mg) subjected to SEC on Sephadex LH-20 column (2.1×67 cm) and MeOH: EtOAC (2:1) was used as solvent to obtain compound 4 (6.5 mg), 5 (3.8 mg) and 6 (9.5 mg). For further purification all compounds were applied to a Sephadex-LH20 CC (1.2×55 cm) eluted with methanol separately. 

*Spectral data of isolated compounds*

Luteolin 1: UV λ _max _nm MeOH: 345.5, 308, 284, 260sh; + AlCl3: 422, 307sh, 286; + AlCl3/HCl: 384, 350, 318sh, 307, 286; + NaOAC : 390, 307, 289 ; + NaOAC/H3BO3 : 430sh, 367, 370, 289; ^1^H NMR (400MHz, DMSO-d_6_): δ 7.46 (1H, *dd*, *J*=8.4, 2.0 Hz, H-6'), 7.00 (1H, *d*, *J*=8.4 Hz, H-5'), 7.52 (1H, *d*, *J*=2.0 Hz, H-2'), 6.57 (1H, *s*, H-3), 6.54 (1H,* d*, *J*=2 Hz, H-8), 6.25 (1H, *d*, *J*=2 Hz, H-6): ^13^C NMR(DMSO-d_6_): δ 182.9 (C-4), 165.2 (C-7), 165.4 (C-2), 162.3 (C-5), 158.5 (C-9), 150.1 (C-4'), 146.1 (C-3′), 122.8 (C-6′), 121.4 (C-1′), 115.9 (C-5'), 113.2 (C-2'), 102.9 (C-3), 102.9 (C-10), 99.2 (C-6), 94.1 (C-8).

Quercetin-7-O-glucoside 2: UV λ _max _nm MeOH: 369, 270sh, 250; + AlCl3: 441, 340sh, 270; + AlCl3/HCl: 430, 368sh, 292sh, 266; + NaOMe: 423, 270, 267sh, 246; + NaOAC : 256, 386 ; + NaOAC/H3BO3 : 254, 420; EIMS: m/z %: 302[M-glucose]^+^(100), 285 [M-OH]^+^(12), 273 [M-COH]^+^(8), 193 [M-B]^+^(12), 153[A1+H]^+^(27), 137[B2]^+^(32), 105[B1-COH]^+^(34); ^1^H NMR (400MHz, DMSO-d_6_): δ 7.56 (1H, *dd*, *J*=8.4, 2.4 Hz, H-6'), 7.74 (1H, *d*, *J*=2.4 Hz, H-2'), 6.91 (1H, *d*, *J*=8.4 Hz, H-5'), 6.77 (1H,* d*, *J*=2 Hz, H-8), 6.42 (1H, *d*, *J*=2 Hz, H-6), 5.08 (1H, *d*, *J*=7.6 Hz, H-1''), 3.5- 4.5 (5H, *m*, H-2''-6''); ^13^C NMR(DMSO-d_6_): δ 174.9 (C-4), 161.6 (C-7), 159.3 (C-5), 157.2 (C-9), 146.9 (C-4'), 146.5 (C-2), 144.0 (C-3'), 141.5 (C-3), 135.0 (C-1′), 120.7 (C-6′), 118.4 (C-2′), 114.5 (C-5'), 103.9 (C-10), 100.0 (C-1''), 98.8 (C-6), 98.0 (C-8), 76.1 (C-5''), 75.4 (C-3''), 72.1 (C-2''), 68.5 (C-4''), 59.6 (C-6''). 

Kaempferol-7-O-glucoside 3: UV λ _max _nm MeOH: 367, 298sh, 267, 255; + AlCl3: 425, 345, 293sh, 266; + AlCl3/HCl: 425, 345, 293sh, 265; + NaOMe: dec; + NaOAC : 325sh, 380, 325sh, 266 ; + NaOAC/H3BO3 : 364, 256; ^1^H NMR (400MHz, DMSO-d_6_): δ 7.93 (2H, *d*, *J*=8.0 Hz, H-2',6'), 6.88 (2H, *d*, *J*=8.0 Hz, H-3',5'), 6.46 (1H, *d*, *J*=2.0 Hz, H-8), 6.20 (1H,* d*, *J*=2.0 Hz, H-6). 

Kaempferol 4: UV λ _max _nm MeOH: 365, 320sh, 295sh, 266, 255; + AlCl3: 425, 330sh, 300sh, 272; + AlCl3/HCl: 425, 330sh, 300sh, 272;+ NaOMe: dec; + NaOAC : 390, 302, 268 ; + NaOAC/H3BO3 : 370, 320sh, 295sh, 265; ^1^H NMR (400MHz, DMSO-d_6_): δ 8.15 (2H, *d*, *J*=8.9 Hz, H-2',6'), 6.99 (2H, *d*, *J*=8.9 Hz, H-3',5'), 6.46 (1H, *d*, *J*=1.9 Hz, H-8), 6.27 (1H,* d*, *J*=1.9 Hz, H-6); ^13^C-NMR(DMSO-d_6_): δ 176.19 (C-4), 163.8 (C-7), 160.7 (C-5), 160.44 (C-4'), 156.75 (C-9), `149.0 (C-2), 136.19 (C-3), 129.52 (C-2′, 6′), 131.72 (C-1′), 115.45 (C-3', 5′), 102.56 (C-10), 98.05 (C-6), 93.5 (C-8). 

Apigenin 5: UV λ _max _nm MeOH: 336, 284, 267.5; + AlCl3: 388, 345sh, 301, 276, 219; + AlCl3/HCl: 387, 343, 300, 276, 217; + NaOMe: 394, 318, 274, 214; + NaOAC : 359, 305, 272; + NaOAC/H3BO3 : 336, 268; EIMS: m/z %: 270[M-glucose]^+^(100), 152[A1](25), 121[B2](36), 118[B1](25); ^1^H NMR (400MHz, DMSO-d_6_): δ 7.76 (2H, *d*, *J*=8.4 Hz, H-2',6'), 6.95 (2H, *d*, *J*=8.4 Hz, H-3',5'), 6.51 (1H, *s*, H-3), 6.48 (1H, *s*, H-8), 6.24 (1H,* s*, H-6).

Apigenin-7-O-glucoside 6: UV λ _max _nm MeOH: 332, 268; + AlCl3: 385, 347, 299, 276; + AlCl3/HCl: 382, 341, 299, 277; + NaOMe: 386, 300, 279, 265; + NaOAC : 397sh, 341, 267; + NaOAC/H3BO3 : 336, 266, 256sh; EIMS: m/z %: 270[M-glucose]^+^(100), 152[A1](18), 120[B2-H](25), 117[B1-H](18); ^1^H NMR (400MHz, DMSO-d_6_): δ 7.84 (2H, *d*, *J*=8.0 Hz, H-2',6'), 6.94 (2H, *d*, *J*=8.0 Hz, H-3',5'), 6.72 (1H, *s*, H-3), 6.66 (1H, *s*, H-8), 6.44 (1H,* s*, H-3), 5.00 (1H, *d*, *J*=7.2 Hz, H-1''), 3.4- 4.5 (5H, *m*, H-2''-6'').

## Results and Discussion

The antimicrobial effects of different fractions of *S. securidaca* seeds was demonstrated in Table 1. The Petroleum ether fraction only inhibited the growth of *S. aureus *and *P. aeruginosa *with inhibition zone diameter of 7.5 -12 mm. The chloroform fraction showed inhibitory effect only against *S. aureus *with inhibition zone of 9.5 -12 mm diameter. The methanol fraction showed no antimicrobial activity. The largest zones of inhibition were observed for petroleum ether fraction against *P. aeruginosa *and chloroform fraction against *S. aureus* (each 100 µg/mL). All fractions exhibited no antifungal activities. 

**Table 1:** Antimicrobial activity of *S. securidaca* seed different fractions by cup-plate method.

Inhibition zone diameter(mm)						Concentration mg/mL	Sample
CA	BS	KP	EC	PA	SA		
-	-	-	-	12	10	100	Petroleum ether Fraction
-	-	-	-	10	8	50	
-	-	-	-	9.5	7.5	25	
-	-	-	-	-	-	12.5	
-	-	-	-	-	12	100	Chloroform Fraction
-	-	-	-	-	10	50	
-	-	-	-	-	9.5	25	
-	-	-	-	-	-	12.5	
-	-	-	-	-	-	100	Methanol Fraction

SA:* S. aureus, *PA: *P. aeruginosa,* EC: *E. coli,* KP: *K. pneumoniae,* BS: *B. subtilis, *CA: *C. albicans, *-: no effect

The phytochemical analysis of *S. securidaca* showed existence of flavonoids, coumarins and cardiac glycosides (14-16). Some flavonoids of *S. securidaca* have been shown potent cytotoxicity by MTT assay against three Human cancer cell lines: colon carcinoma (HT-29), breast ductal carcinoma (T47D) and colorectal adenocarcinoma (Caco-2) (17).

There were reports for antimicrobial effects of some cardenolides (18, 19), and the antibacterial activity of *S. securidaca* may be due to existence of this class of compounds. 

*Rosa damascena *extract showed good antibacterial activities against *B. cereus*, *S. aureus*, *and S. epidermidis* as Gram positive bacteria and *P. aeruginosa* as Gram negative bacteria with MICs (Minimum Inhibitory Concentration) 70, 140, 560 and 140 µg/mL, respectively. It was inactive against other microorganisms with MICs of >1000 μg/mL. The inhibition zone diameter of *R. damascena* extract against *S. aureus* and *S. epidermidis* is more than Gentamycin as positive control (5 µg/mL) (Table 2). 

**Table 2:** Antimicrobial activity of *R. damascena *and *T. disciforme* extract by cup-plate method.

	Concentration mg/mL	Inhibition zone diameter(mm)							
		BC	BS	SA	SE	EC	PA	AN	CA
*R. damascena *Extract	64	14	-	24	18	-	12	-	-
	32	13.4	-	23	16	-	11	-	-
	16	13.3	-	18	15	-	10.3	-	-
	8	13.2	-	16	13	-	10	-	-
	4	13	-	13	-	-	9.6	-	-
	2	11	-	10	-	-	9	-	-
	1	10	-	-	-	-	-	-	-
	0.5	-	-	-	-	-	-	-	-
*T. disciforme* Extract	64	-	-	14	12	-	-	-	-
	32	-	-	10.2	10	-	-	-	-
	16	-	-	10	-	-	-	-	-
	8	-	-	-	-	-	-	-	-
	4	-	-	-	-	-	-	-	-
	2	-	-	-	-	-	-	-	-
	1	-	-	-	-	-	-	-	-
	0.5	-	-	-	-	-	-	-	-
Gentamycin	5	25	18	18	12	18	19	-	-
Nystatin	50	-	-	-	-	-	-	23	25

BC: *B. cereus*, BS: *B. subtilis, *SA:* S. aureus, *SE: *S. epidermidis*, EC: *E. coli,* PA: *P. aeruginosa,* AN: *A. niger,* CA: *C. albicans, *-: no effect

A previous investigation showed the MIC of butanol extract of *R. damascena *receptacles against *Salmonella* *typhimurium *and *Bacillus cereus* were 62.5 and 250 μg/mL, respectively.

Aqueous extract of *R. damascena *receptacles were inhibited* Candida albicans *and Methicillin-resistant *S. aureus* with MIC of 125 and 500 μg/mL (20). It is obvious that antimicrobial potential of crude extract of *R. damascena *against *B.* *cereus* was more than that of butanol extract. Another study demonstrated fresh and spent flower extracts of *R. damascena* showed the strongest effects against *Salmonella enteritidis* and *Mycobacterium smegmatis*. Both extracts were not effective against *E. coli* (9).

*Tripleurospermum disciforme *extract showed antimicrobial effects only against *S. aureus* and *S. epidermidis *with MICs 112 and 224 μg/mL, respectively. It was inactive against the other microorganisms (Table 2). Another study reported the essential oil of *T. disciforme* was effective on *Staphylococcus subtilis *and *Bacillus cereus *with MICs 4 µL/mL and on *Citrobacter amalonaticus* with MIC 22 µL/mL (21). Methanol extract of *T. disciforme* were not exhibited antiproliferative activity by using the MTT assay against: A549, human lung adenocarcinoma; MCF7, human breast adenocarcinoma; HepG2, hepatocellular carcinoma; HT-29, human colon carcinoma and one normal cell line MDBK, bovine kidney (22). 

**Figure 1:**
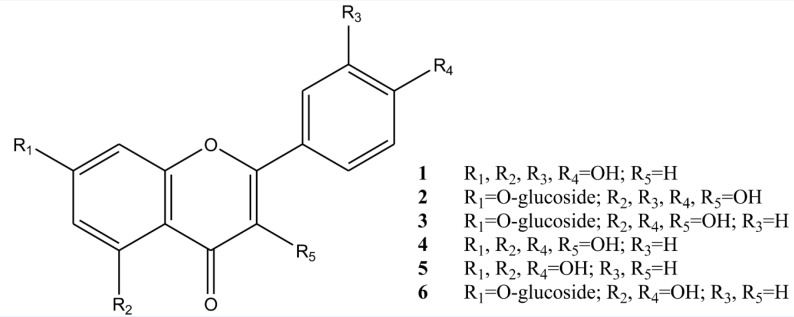
Chemical structure of isolated compounds of *Tripleurospermum disciforme.*

There was only one report about phytochemical investigation on flowers extract of *T. disciforme *which demonstrated isolation of a new dioxaspiran derivative (23). In our study, six flavonoids were isolated from *T. disciforme*: Luteolin, Quercetin-7-O-glucoside, Kaempferol, Kaempferol-7-O-glucoside, Apigenin and Apigenin-7-O-glucoside. The isolated compounds were identified using different spectroscopic methods (Figure 1).

Flavonoids act as antimicrobial agents in different ways including direct antibacterial activity, synergism with antibiotics and suppression virulence (24). Many researchers investigated the antibacterial activity of flavonoids (25), for example, it can be mentioned the antibacterial activities against *Propionibacterium acnes *by kaempferol and quercetin (26), inhibitory effects of apigenin against *S. typhi*, *Proteus mirabilis* and *P. aeruginosa* (27) and selective toxicity of apigenin and luteolin against *S. aureus* including the MRSA and methicillin-sensitive *S. aureus* strains (28, 29).

## Conclusion

In conclusion, *Rosa damascena* and *Tripleurospermum disciforme* have shown antimicrobial effects against *Staphylococcus* strains. These results confirmed the folklore consumption of distilled water of *R. damascena* as tonic and face cleanser and fume of *T. disciforme* as tonic and disinfectant for treatment of acne. Because of antibiotic resistance of *S. aureus*, these two herbs can be used in health and beauty products for treatment of skin disorders especially acne in teenagers. 
